# Dynamic Changes of DNA Methylation and Transcriptome Expression in Porcine Ovaries during Aging

**DOI:** 10.1155/2019/8732023

**Published:** 2019-10-30

**Authors:** Xiaoyu Xi, Qin Zou, Yingying Wei, Yan Chen, Xue Wang, Daojun Lv, Peilin Li, Anxiang Wen, Li Zhu, Guoqing Tang, Jideng Ma, Mingzhou Li, Xuewei Li, Yanzhi Jiang

**Affiliations:** ^1^Department of Zoology, College of Life Science, Sichuan Agricultural University, Ya'an, Sichuan 625014, China; ^2^Sichuan Weimu Modern Agricultural Science and Technology Co., Ltd., Chengdu, Sichuan 611536, China; ^3^Institute of Animal Genetics and Breeding, College of Animal Science and Technology, Sichuan Agricultural University, Chengdu, Sichuan 611130, China

## Abstract

The biological function of human ovaries declines along with aging. To identify the underlying molecular changes during ovarian aging, pigs were used as model animals. Genome-wide DNA methylation and transcriptome-wide RNA expression analyses were performed via high-throughput sequencing of ovaries from young pigs (180 days, puberty stage of first ovulation) and old pigs (eight years, reproductive exhaustion stage). The results identified 422 different methylation regions between old and young pigs; furthermore, a total of 2,243 mRNAs, 95 microRNAs, 248 long noncoding RNAs (lncRNAs), and 116 circular RNAs (circRNAs) were differentially expressed during both developmental stages. Gene ontology analysis showed that these genes related to different methylation and expression are involved in the ovarian aging cycle. Specifically, these are involved in cell apoptosis, death effector domain binding, embryonic development, reproduction and fertilization process, ovarian cumulus expansion, and the ovulation cycle. Multigroup cooperative control relationships were also assessed, and competing endogenous RNA (ceRNA) networks were constructed in the ovarian aging cycle. These data will help to clarify ovary age-associated potential molecular changes in DNA methylation and transcriptional patterns over time.

## 1. Introduction

The major challenges in women's reproductive health are the reduction of reproductive performance along with aging; moreover, oocyte quantity and oocyte quality are closely related to the reduction of follicular reserve in the ovary [1]. The decrease of follicular reserve in the ovary is nonlinear and accelerated with age [2–4]. This leads to near-complete exhaustion by a mean age between 51 and 52 years, which is defined as menopause [2]. Ovarian aging is affected by numerous factors and has been particularly linked to genetics [5, 6]. In conclusion, the heredity record of normal reproduction and numerous pathologies like premature ovarian insufficiency (POI) and polycystic ovary syndrome (PCOS) have been emphasized [6, 7].

The concept of epigenetics related to heritable changes in chromatin structure and gene expression does not involve changes of DNA sequence. The known classes of epigenetic modification are DNA methylation, histone modification, and the synthesis of noncoding RNA, including that of microRNAs (miRNAs) and long noncoding RNAs (lncRNAs) [8, 9]. Furthermore, these three epigenetic mechanisms are in fact forming a network [9]. “Ovarian epigenetics” is a new field that has uncovered stimulating revelations. Recent research has documented that DNA methylation plays a key role in the regulation of ovarian cancer and ovarian diseases such as PCOS and POI [10, 11]. Moreover, several noncoding RNAs such as miRNAs and lncRNAs are crucial for the regulation of ovarian physiology [12–16]. However, these results mainly elucidate the epigenetic mechanism in ovarian health-associated phenotypes. For natural ovarian aging, epigenetic mechanisms in the ovarian context have been studied far less, due to the timing of menarche and menopause. Importantly, epigenetic modifications during the natural aging of ovaries are a perfect opportunity to understand the health-related phenotypes of ovaries. The reason is that these studies not only pointed out the mechanisms of ovarian aging but also elucidated the complex interaction networks of different ovarian phenotypes.

It is both difficult and unethical to investigate the ovarian mechanisms in women. While laboratory rodents (e.g., mice) are useful models for biomedical research, they offer comparatively limited use for the study of ovarian changes in mammals, due to their small body size and extremely short ovarian cycle [17]. The big animal models such as equine, bovine, and ovine have been confirmed to be valuable and useful for investigation on the ovarian function in women [18]. Therefore, pigs can be a valuable model to study human ovarian aging or disease due to its similar cycle length, luteinizing hormone (LH) receptor location and its function, length of ovulation, and LH surge [19] as well as their anatomical, physiological, and biochemical similarities to humans [20]. In fact, a number of researchers have identified pig as an ideal model system to investigate the effects of metabolic syndrome and obesity in relation to the function and steroidogenesis of ovaries [21, 22].

Female natural ovarian aging is defined between the two time points (menarche and menopause) in a woman's life that open and close the reproductive system. The median ages for menarche and menopause are about 14 and 50 years, respectively [3]. Both time points are central to ovarian function, and several recent genome-wide association studies (GWAS) explained the genetic background of traits, both for the timing of menarche [23–25] and menopause [26–28]. Correspondently, the puberty stage of the first ovulation and the reproductive exhaustion stage are two key points in the ovarian cycle. The median ages of the sow puberty stage of first ovulation and reproductive exhaustion stage are about 180 days and eight years, respectively [29, 30].

To identify the potential molecular changes during natural ovarian aging, the pig was used as a model animal. Genome-wide DNA methylation and transcriptome-wide RNA expression analyses were performed via high-throughput sequencing of ovaries from young (180 days, puberty stage of first ovulation) and old (eight years, reproductive exhaustion stage) sows. This enabled the determination of a number of differentially methylated and expressed genes or regulatory elements and previously unclear multigroup cooperative control networks in the ovarian aging cycle. These data will help to explain ovary age-associated changes in DNA methylation and transcriptional patterns over time.

## 2. Materials and Methods

### 2.1. Animal Material and Sample Preparation

Four healthy female Yanan pigs were utilized in this experiment. The Yanan pig is an indigenous Chinese pig breed, which emerged in the hilly areas of western Sichuan Province in the past. Due to the poor growth performance and carcass composition, it is endangered by extinction. These four pigs included two eight-year-old sows at the reproductive exhaustion stage and two 180-day young sows at the puberty stage of first ovulation. These pigs had no direct or collateral blood relationship during the last three generations. Piglets were weaned at the age of 28 ± 1 days. An initial diet started from the 30^th^ to the 60^th^ day after weaning and contained 3.40 Mcal·kg^−1^ of metabolizable energy with 20.0% crude protein (11.5 g/kg lysine). From the 61^st^ to the 120^th^ day, pigs were given a diet containing 14.0 MJ/kg of metabolizable energy comprising 18% of crude protein (9.0 g/kg lysine). From the 121^st^ day, they received a dietary metabolizable energy and crude protein (8.0 g/kg lysine) of 13.5 MJ/kg and 16%, respectively. Pigs were allowed to access water and food ad libitum and were kept under similar conditions. The night before slaughtering, pigs were not allowed to feed and were given 2 h rest after transportation, then stunned electrically at 90 V and 50 Hz for 10 s, and exsanguinated to ameliorate pain. All animal experiments and procedures were conducted according to the Regulations for the Administration of Affairs Concerning Experimental Animals (Ministry of Science and Technology, China, revised in June 2004) and were approved by the Institutional Animal Care and Use Committee in College of Animal Science and Technology, Sichuan Agricultural University, Sichuan, China, under permit no. SKY-S20150804. All research animals were obtained from Sichuan Weimu Modern Agricultural Science and Technology Co., Ltd., Chengdu, Sichuan 611536, P. R. China. Ovary tissues were rapidly collected from every carcass and directly frozen in liquid nitrogen after separation. All collected samples were stored at −80°C until the extraction of DNA and total RNA.

### 2.2. Whole Genome Bisulfite Sequencing (WGBS) and Data Analysis

Bisulfite treatment of 50–100 ng of purified genomic DNA was performed using the Zymo EZ DNA Methylation Lightning Kit. 50 to 100 ng of purified genomic DNA was treated with Zymo Lightning Conversion Reagent for 8 min at 98°C in a thermal cycler and then for 60 min at 54°C. Bisulfite-treated DNA was purified via spin column and was used to build a sequencing library with the help of the EpiGnome™ Kit (Epicentre). In this process, bisulfite-treated single-stranded DNA was arbitrary primed using a polymerase with the ability to read uracil nucleotides, to manufacture DNA with a particular sequence tag. 3′ ends of the newly manufactured DNA strands were tagged with a 2^nd^ specific sequence, resulting in ditagged DNA molecules at both their 5′ and 3′ ends with identified sequence tags. Then, these tags were used to add Illumina adapters P7 at the 5′ and P5 at the 3′ end of the original DNA strand by polymerase chain reaction (PCR). Only the complement to the original bisulfite-treated DNA was used as sequencing template; therefore, the resulting read always had a similar sequence like the original bisulfite-treated strands. Then, bisulfite genome DNA libraries were sequenced by the Illumina Hiseq 4000 platform with 150PE reads.

Reads were alimented to the *Suscrofa* 10.2 with Bismark tools after quality filtering [31]. Bowtie2 was called by Bismark when mapped reads to reference. Parameters were multiseed length of 20 bp with 0 mismatches and minimum alignment score function L, 0, and −0.2 [32]. For 150 bp reads, this would mean a minimum alignment score of −30 before an alignment would become invalid. This is approximately equal to four mismatches or ∼4 InDels of 1–4 bp in the read [31]. After reads were alimented, SAMtools were applied to deal with the bam file out form Bowtie2 [32]. CpG island locus information was downloaded from the UCSC genome browser, and CpG island methylation level of each sample was calculated with bedtools [33]. To identify differentially methylated regions between both stages, the R package DSS was used to call differentially methylated loci with p.threshold <0.001 first and then different methylation regions (DMR) with delta <0.1 with p.threshold < 0.001 [34, 35]. The function gene ontology (GO) of the DMR target gene was enriched by topGO [36]. At last, other data statistics and visualizations were conducted in R and Python scripts.

### 2.3. RNA Sequencing and Data Analysis

A total amount of 5 *μ*g RNA of each sample was used as input material for RNA sample preparations. The rRNA-depleted RNA was used to generate sequencing libraries by NEBNext® Ultra™ Directional RNA Library Prep Kit for Illumina® (NEB, USA) according to the instructions of the manufacturer, and the Agilent Bioanalyzer 2100 system was used to assess library quality. After cluster generation, Illumina HiSeq 4000 was used for the sequencing of libraries and paired-end reads were generated at 150 bp. After removing the adapter, ploy-N, and low-quality reads from raw data, clean reads were obtained. These clean reads were aligned to the Ensemble (*Susscrofa* 10.2) using TopHat2 (v2.0.14) with default parameters [37].

StringTie software was used to assemble the mapped reads per sample [38], which worked in at least one of both replicates. The obtained transcripts were blasted (*e* value = 1*e* − 10) to Ensemble, and mapped transcripts were directly described as known lncRNA or mRNA. Salmon (v0.6.0) was used to calculate transcripts per million (TPMs) of both lncRNAs and mRNAs per sample [39]. Then, coding potential calculator (CPC, 0.9) and Pfam Scan v1.5 were used to examine the transcript's coding potential [40, 41]. Transcripts predicted with coding potential were filtered out, and the transcripts without coding potential were regarded as candidate set of novel lncRNAs.

### 2.4. Small RNA Sequencing and Data Analysis

A total amount of 5 *μ*g RNA of each sample was used as input material for library preparation of small RNA. Sequencing libraries were created using NEBNext® Multiplex Small RNA Library Prep Set for Illumina® (NEB) according to the instructions of the manufacturer. In every sample index, codes were added to attribute sequences. The Agilent Bioanalyzer 2100 system was used to analyze library quality. The clustering of index-coded samples was performed on a cBot Cluster Generation system using TruSeq SR Cluster Kit v3-cBot-HS (Illumina), following the manufacturer's recommendations. After the generation of clusters, the preparations for library sequencing were conducted on an Illumina MiSeq platform and single-end reads were generated at 50 bp.

miRBase 21 was used as reference, and the software mirdeep2 was used to obtain the potential miRNA and to predict novel miRNA [42]. The miRanda (v3.3a, default parameters) and cutoffs (score *S* ≥ 140 and energy *E* ≤ −20.0) were used to predict the miRNA target [43]. Then, miRNA expression was assessed by TPMs through the following criteria: normalization formula: normalized expression = mapped read count/total reads *∗* 10,00,000.

### 2.5. Differential Expression Analysis and Gene Ontology Enrichment Analysis

Differentially expressed mRNAs, lncRNAs, miRNAs, and circRNAs were found by using the edgeR package [44]. A differential expression *P* value <0.05 and fold change >2 were assigned as differentially expressed in different comparisons. GO enrichment was performed by TopGO [36]. Other data statistics and visualizations were performed by self-written R scripts.

### 2.6. Network with MicroRNA Response Element (MRE) and Coexpression Network

The network of RNAs with MRE was established by target prediction of miRNA-mRNA, miRNA-lncRNA, and miRNA-circRNA with bioinformatics and visualized with the R package igraph. The coexpression network of mRNA, miRNA, lncRNA, and circRNA (circular RNAs) was created based on Pearson's correlation coefficient of expression. Pearson's correlation coefficient of 0.85 between two RNAs was considered relevant for network construction. A *P* value below 0.05 was considered statistically significant.

### 2.7. Bisulfite Sequencing PCR (BSP)

The primers for BSP were designed by primer software V5.0 (Supplementary [Supplementary-material supplementary-material-1]). The inspected DNA (bisulfite conversion) was treated using the EpiTect Fast DNA Bisulfite Kit (Qiagen) according to the manufacturer's protocols. PCR product was purified using the UNIQ-10 Spin Column DNA Gel Extraction Kit for PAGE (Sangon) and was then cloned with the pGM-T Fast Cloning Kit with competent cell (Tiangen). Ten effective clones were selected per gene, and then, an ABI 3730 DNA sequencer was used for sequencing. DNAMAN 7.0 (Lynnon Biosoft, USA) was used to analyze all sequences.

### 2.8. Quantitative Real-Time PCR (q-PCR)

Total RNAs were extracted from ovaries using the HiPure Universal RNA Mini Kit (Magen, China) and reversely transcribed into cDNA using the oligo (dT) and random 6-mer primers, provided by the PrimeScript RT Master Mix kit (TaKaRa). The q-PCR was performed using a standard SYBR Premix Ex Taq kit (Takara, Dalian, China) on a Bio-RadCFX96 Real-Time PCR Detection system (Bio-Rad, Hercules, CA, USA) following the manufacturer's directions. Three endogenous control genes, glyceraldehyde-3-phosphate dehydrogenase (GAPDH), *β*-actin (ACTB), and small nuclear RNA (U6 snRNA), were used in this assay. The 2^−ΔΔCt^ method was used to determine the expression levels of objective mRNAs, miRNAs, lncRNAs, and circRNAs [45]. These primers are shown in Supplementary [Supplementary-material supplementary-material-1].

## 3. Results

### 3.1. Summary of Whole Genome Bisulfite Sequencing (WGBS) Data

**Figure 1:**
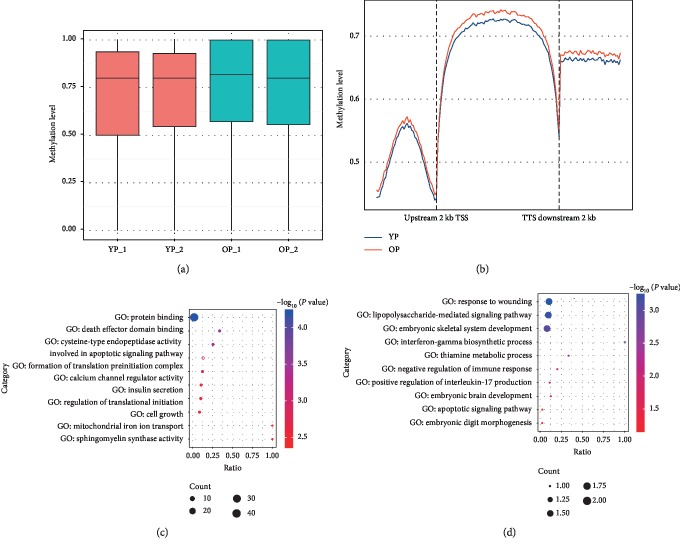
DNA methylation associated with ovarian aging. (a) DNA methylation levels between both ovarian development stages. YP-1and YP-2 represent sample 1 and sample 2 from young sows at the puberty stage, respectively; OP-1 and OP-2 represent sample 1 and sample 2 from old sows at the reproductive exhaustion stage, respectively. (b) Distribution of CG methylation reads on and around the gene body region. Abbreviations: TSS, transcription start site; TTS, transcription termination site. Gene ontology (GO) function enrichment of the (c) hypermethylated genes and (d) hypomethylated genes related to the different methylation regions (DMRs).

To assess the changes in DNA epigenetic marks by aging that occurred during ovarian development, DNA methylation profiles were determined using WGBS between old pig (OP) ovarian tissues at reproductive exhaustion stage and young pig (YP) ovarian tissues at the puberty stage of first ovulation. Approximately 951,440,304 clean reads were obtained from the four ovarian samples, which provided about 30x sequencing depth. After removing unclear reads from clean reads, approximately 61–68% reads per sample were uniquely aligned to Ensemble *Susscrofa* 10.2 (Supplementary [Supplementary-material supplementary-material-1]). The global methylation levels were 70.8% for YP and 72.9% for OP, respectively. There was no significant difference between the two ovarian development stages (Figure 1(a)), although a genome-wide loss of DNA methylation was found in response to age-related epigenetic modification [46]. Analysis of the sequence context of cytosines showed methylation that occurred in the CpG and non-CpG (CHG and CHH) context in most chromosomal regions in each group. Differential methylation between both ovarian developmental stages mostly occurred at CpG context regions (Supplementary [Supplementary-material supplementary-material-1] and Supplementary [Supplementary-material supplementary-material-1]).

To understand the preferential location of the CG methylation on and surrounding the gene body region (GBR), the metagene profiles of CG methylation were investigated in the entire pig genome of the ovarian tissue. Both GBR and adjacent intergenic regions were heavily methylated; however, slightly higher levels of methylation were recorded in gene bodies than in neighboring intergenic regions (Figure 1(b)), which have similar distribution to those of human primordial germ and prenatal germline cells [47, 48] as well as pig skeletal muscle cells [49].

To examine the dynamics of methylation on a global scale, correlation analysis between genomic features and methylation levels was conducted. Negatively correlated methylation levels were observed across chromosomes with chromosomal length (Pearson's *r* = −0.636, *P* = 1.844 × 10^−9^), and a positive correlation was observed with the GC content (the percentage of guanine and cytosine, *r* = 0.903, *P* < 2.2 × 10^−16^), gene number (being calculated for a 1 Mb window in chromosomes, *r* = 0.398, *P* < 2.2 × 10^−16^), CpG_o/e_ (the ratio between the observed and expected numbers of CpG sites, *r* = 0.792, *P* < 2.2 × 10^−16^), and repeat region density (being calculated for a 1 Mb window in chromosomes, *r* = 0.126, *P* < 2.2 × 10^−16^) (Supplementary [Supplementary-material supplementary-material-1]). These results are similar with previous reports [50, 51]. Among these genomic features, the GC content and CpG_o/e_ showed the strongest correlations with the methylation level. The gene density also showed a moderate correlation with the methylation level, which may be due to the higher GC content examined in gene regions, which contributed to this (Figure 1(b)), and suggests a possible role of methylation dynamics in the gene transcription regulation [52].

### 3.2. Differential DNA Methylation Associated with Ovarian Aging

To further discover the differential CG methylation across ovarian aging, different methylation regions (DMRs) were identified between OP and YP. As a result, 422 DMRs were identified between both developmental stages and 303 of these DMRs were upmethylated while 119 DMRs were downmethylated in OP compared to YP (*P* < 0.001, Supplementary [Supplementary-material supplementary-material-1]). The dynamical DMR level at both ovarian developmental stages suggested that DNA methylation may play a crucial role in ovarian aging. Furthermore, 146 of these 422 DMRs overlapped at gene body regions while only 12 of these DMRs were located in the gene promoter regions (Supplementary [Supplementary-material supplementary-material-1]). This agreed with the preferential location of the CG methylation on the gene body regions (Figure 1(b)). Previous research also reported that more DMRs are enriched in gene bodies than in promoters of porcine skeletal muscle [51].

To characterize the role of genes associated with these DMRs, GO enrichment was performed. The results showed that hypermethylated genes related to DMRs were involved in numerous cellular functions like protein binding, death effector domain binding, and cysteine-type endopeptidase activity involved in the apoptotic signaling pathway (Figure 1(c) and Supplementary [Supplementary-material supplementary-material-1]). Notably, hypomethylated genes that were related to DMRs depicted significant enrichment for different processes associated to embryonic skeletal/brain system development, embryonic digit morphogenesis, negative regulation of immune response, and apoptotic signaling pathway. For instance, the CASP8- and FADD-like apoptosis regulator gene (*CFLAR*) is an important molecule of the innate immune regulation network and a key suppressor of steatohepatitis [53] and was significantly upmethylated in old pigs. However, the Meckel syndrome Type 1 gene (*MKS1*) in Meckel–Gruber syndrome causes developmental malformations and cilia defects [54] and was significantly downmethylated in old pigs. Furthermore, the BSP results for methylation levels of the two genes were in accordance with the WGBS data between OP and YP (Supplementary [Supplementary-material supplementary-material-1]A).

### 3.3. DNA Methylation and Gene Expression in Gene Body

The effect of methylation on promoter regions was reported to be an important mechanism in regulating the gene transcription [55]. However, the precise roles of DNA methylation in gene body are yet to be discovered. To search how gene expression affected intergenic methylation, the RNA sequence data were used (Supplementary [Supplementary-material supplementary-material-1]) to correlate DMR-mRNA pairs. A significant negative correlation (*r* = −0.179, *P* = 5.72 × 10^−7^) was found between changes in the methylation levels in the gene bodies and gene expression levels. Similar to these findings, previous methylation analysis in gene bodies described a significantly negative correlation with the expression levels of mRNA [51]. However, previous scientists reported a positive correlation with gene expression levels [56, 57] or no clear relationship patterns [58].

### 3.4. Transcriptome Profiles of Ovarian Aging

To assess transcriptional expression changes during ovarian aging, RNA and small RNA libraries were constructed for OP and YP samples, respectively, and transcriptome-wide profiling (mRNA, miRNA, lncRNA, and circRNA) was determined via high-throughput sequencing. For RNA sequencing libraries, an average of ∼75 million clean reads were obtained from each of the four samples and more than 69% of these reads could be uniquely aligned to the Ensemble *Susscrofa* 10.2 (Supplementary [Supplementary-material supplementary-material-1]). Furthermore, for small RNA sequencing libraries, approximately 10.89–13.50 million clean reads were obtained from each of the four samples and 80.77–90.5% of these reads were uniquely aligned to the Ensemble *Susscrofa* 10.2 (Supplementary [Supplementary-material supplementary-material-1]).

In total, 20,357 mRNAs were identified in these four samples, representing approximately 59.82% of the entire number of transcripts in pigs (Supplementary [Supplementary-material supplementary-material-1]). Moreover, 1,196 miRNAs were identified in these four samples, and 869 potential novel miRNAs out of these miRNAs were detected that did not match the previously reported sequences (Supplementary [Supplementary-material supplementary-material-1]). A total of 4,879 lncRNAs and 7,600 circRNAs were also identified in these four samples (Supplementary [Supplementary-material supplementary-material-1]).

### 3.5. Differentially Expressed Transcriptomes Involved in Ovarian Aging

**Figure 2:**
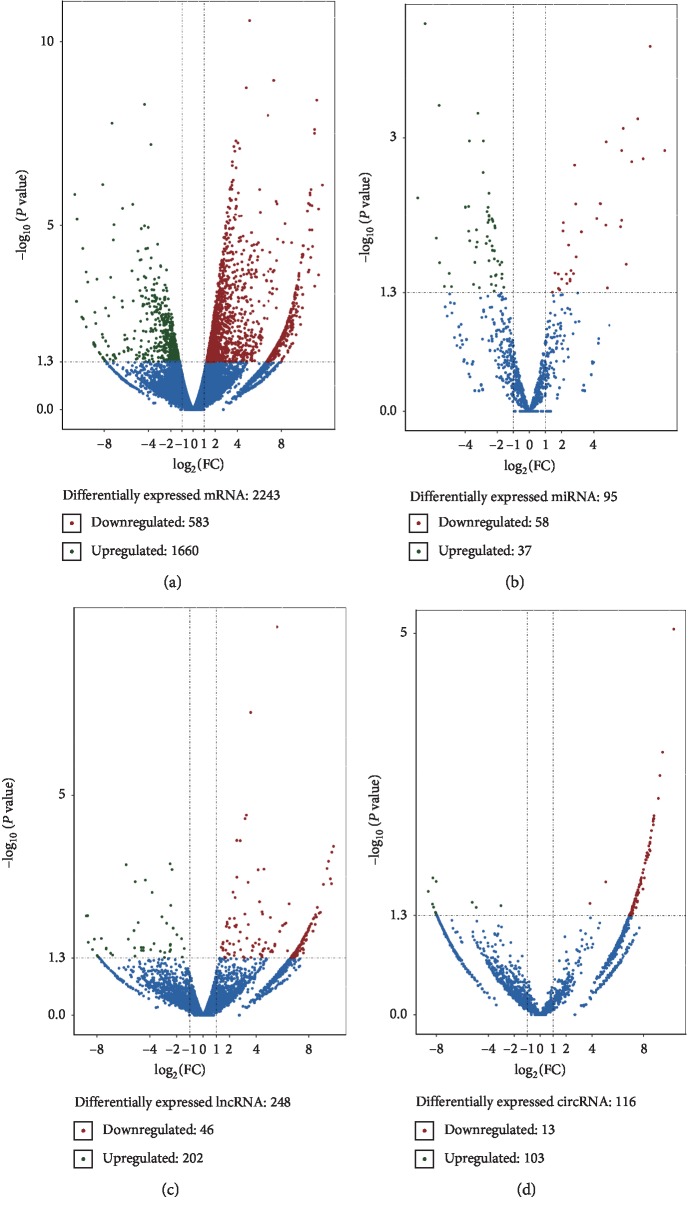
Differentially expressed transcriptome between both ovarian development stages. The *x*-axis indicates log_2_FC, and the *y*-axis indicates the−log_10_*P* value. Criteria of |FC| < 1 and *P* value <0.05 were used to screen differently expressed RNAs. (a) Differentially expressed mRNAs between both ovarian development stages. (b) Differentially expressed miRNAs between both ovarian developmental stages. (c) Differentially expressed lncRNAs between both ovarian developmental stages. (d) Differentially expressed circRNAs between both ovarian developmental stages.

**Figure 3:**
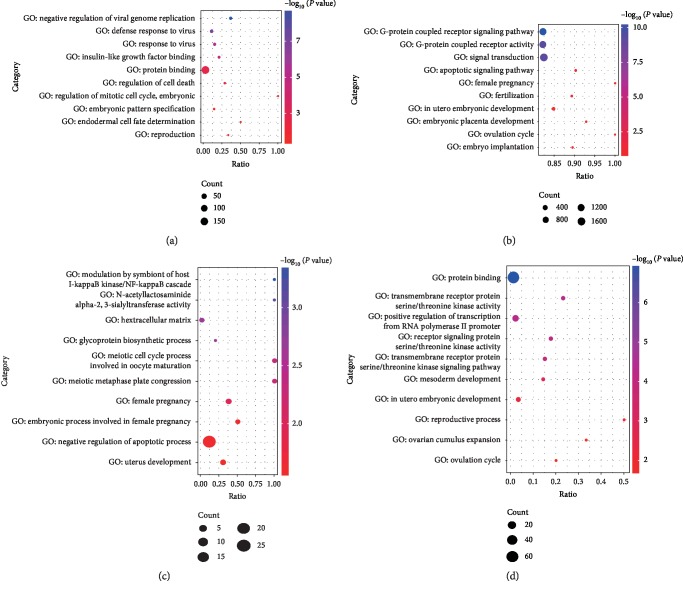
Gene ontology (GO) function enrichment of differentially expressed RNAs between both ovarian development stages. GO analysis of differentially expressed (a) mRNAs, (b) miRNA target genes, (c) lncRNA target genes, and (d) circRNA source genes.

According to the chosen screening criteria (*P* ≤ 0.05), 2,243 differentially expressed genes (DEGs) were found in both stages of ovarian development, which included 1,660 upregulated genes and 583 downregulated genes (Figure 2(a) and Supplementary [Supplementary-material supplementary-material-1]). GO analysis showed that these DEGs were mainly enriched in the extracellular region and involved in a variety of cellular functions including the defense response to virus, regulation of cell death, embryonic pattern specification, and reproduction process (Figure 3(a) and Supplementary [Supplementary-material supplementary-material-1]). For instance, *ISG15* (ISG15 ubiquitin-like modifier) repressed interferon-*α*/*β* overamplification and autoinflammation [59], and the higher expression level of *ISG15* in old pigs suggested that old females were more susceptible to viral infection. Furthermore, SRY-Box 9 (*SOX9*) battles of sexes with Forkhead Box L2 (*FOXL2*), which exert a decisive role during ovary maintenance process and somatic sex reprogramming of adult ovaries to tests [60]. The higher expression level of *SOX9* in OP indicated that the reproductive performance decline in old females.

Ninety-five differentially expressed miRNAs (DEMs) were identified during both stages of ovarian development, and 37 miRNAs of these DEMs were upregulated while 58 miRNAs were downregulated (Figure 2(b) and Supplementary [Supplementary-material supplementary-material-1]). GO analysis showed that the target genes of DEMs are enriched in the G-protein coupled receptor signaling pathway, apoptotic signaling pathway, female pregnancy, fertilization process, embryonic development, and ovulation cycle (Figure 3(b) and Supplementary [Supplementary-material supplementary-material-1]). For example, the upregulated miR-9 plays a key role in the determination of the neural fates in embryonic stem (ES) cell differentiation [61] and has prospective importance in recurrent ovarian cancer as biomarkers [62].

For differentially expressed lncRNAs (DELs) related to ovarian aging, 248 DELs were identified in both ages, which included 202 upregulated DELs and 46 downregulated DELs (Figure 2(c) and Supplementary [Supplementary-material supplementary-material-1]). GO analysis showed that these target genes of DELs were significantly enriched for modulation by symbiont of host I-kappaB kinase/NF-kappaB cascade, meiotic cell cycle process involved in oocyte maturation, female pregnancy, and uterus development (Figure 3(c) and Supplementary [Supplementary-material supplementary-material-1]). Furthermore, 116 differentially expressed circRNAs (DECs) were identified in both ages; 103 circRNAs of these DECs were upregulated while 13 circRNAs were downregulated (Figure 2(d) and Supplementary [Supplementary-material supplementary-material-1]). These DEC source genes were enriched in transmembrane receptor protein serine/threonine kinase activity, in utero embryonic development, reproductive process, ovarian cumulus expansion, and ovulation cycle (Figure 3(d) and Supplementary [Supplementary-material supplementary-material-1]).

### 3.6. Multiomics Coordinated Regulation in Ovarian Aging

To discover the coordinated regulation relationship among DNA methylation and several RNA species during ovarian aging, the number of genes and their percentages were first measured in every potential combination of regulation. Among DNA methylation, mRNA, and miRNA, 9,633 (OP) and 8,845 (YP) genes (representing 38.2% and 34.9% of the entire number of genes in swine genome, respectively) were methylated or simultaneously expressed. Except for each of these three combination groups, the number of genes that were methylated or expressed at the same time decreased and approximately 600 genes were not methylated or expressed simultaneously in OP or YP (Supplementary [Supplementary-material supplementary-material-1]). Similarly, among DNA methylation, lncRNA, and miRNA, 1,465 (OP) and 1,354 (YP) genes (representing 18.1% and 16.7% of the entire gene number in swine genome, respectively) were methylated or simultaneously expressed. The lowest number of genes (175 for OP and 232 for YP) was not methylated or expressed simultaneously (Supplementary [Supplementary-material supplementary-material-1]). The combination regulation relationships of gene transcriptional regulation and gene posttranscriptional regulation were also shown in each chromosome (Supplementary [Supplementary-material supplementary-material-1]). The results indicate that a large number of genes tended toward combination regulation between DNA methylation and transcriptome expression. The utilized combination regulation pattern might be more pronounced in OP. In addition, the DMRs and the differentially expressed transcriptome were screened to identify the overlap between DMRs and DEGs. The results showed that seven DEGs were differentially methylated during ovarian aging, most of which were found in the genebody regions, and only one gene was methylated in the gene promoter region (Supplementary [Supplementary-material supplementary-material-1]). However, no consistent pattern of change was found between methylation and expression levels in these methylated genes in genebody regions. Interestingly, the gene that was methylated in the promoter region was hypomethylated and upexpressed simultaneously during aging in the ovary. These results indicate that changes of methylation occurred in the gene promoter region rather than the genebody region, which might be close to the regulation of the gene expression level during aging.

**Figure 4:**
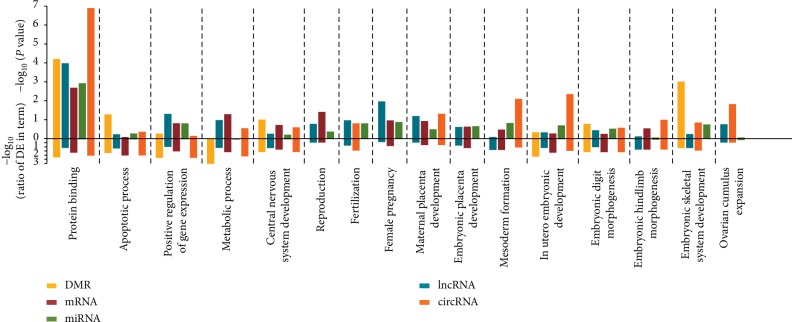
Overlaps of gene ontology (GO) function enrichment among different DNA methylation regions and different expression RNAs. The top different methylation regions (DMRs) and expressed RNAs (miRNAs, mRNAs, lncRNAs, and circRNAs) were both GO enriched. The *x*-axis indicates the RNA class and DMR enriched at GO term, the upper half of *y*-axis indicates the *P* value of enrichment, and the lower half of *y*-axis indicates the ratio of differentially expressed and background of GO term.

To further highlight the potentially coordinated control roles of DNA methylation and several RNA species involved in ovarian aging, GO terms of genes related to DMRs and four differentially expressed RNA species were overlapped. Among DMRs and these differentially expressed RNAs, several intersections of molecular functions and signaling pathways were found that are involved in the ovarian aging cycle such as embryonic development, apoptotic process, reproduction and fertilization, ovarian cumulus expansion, and female pregnancy (Figure 4 and Supplementary [Supplementary-material supplementary-material-1]).

**Figure 5:**
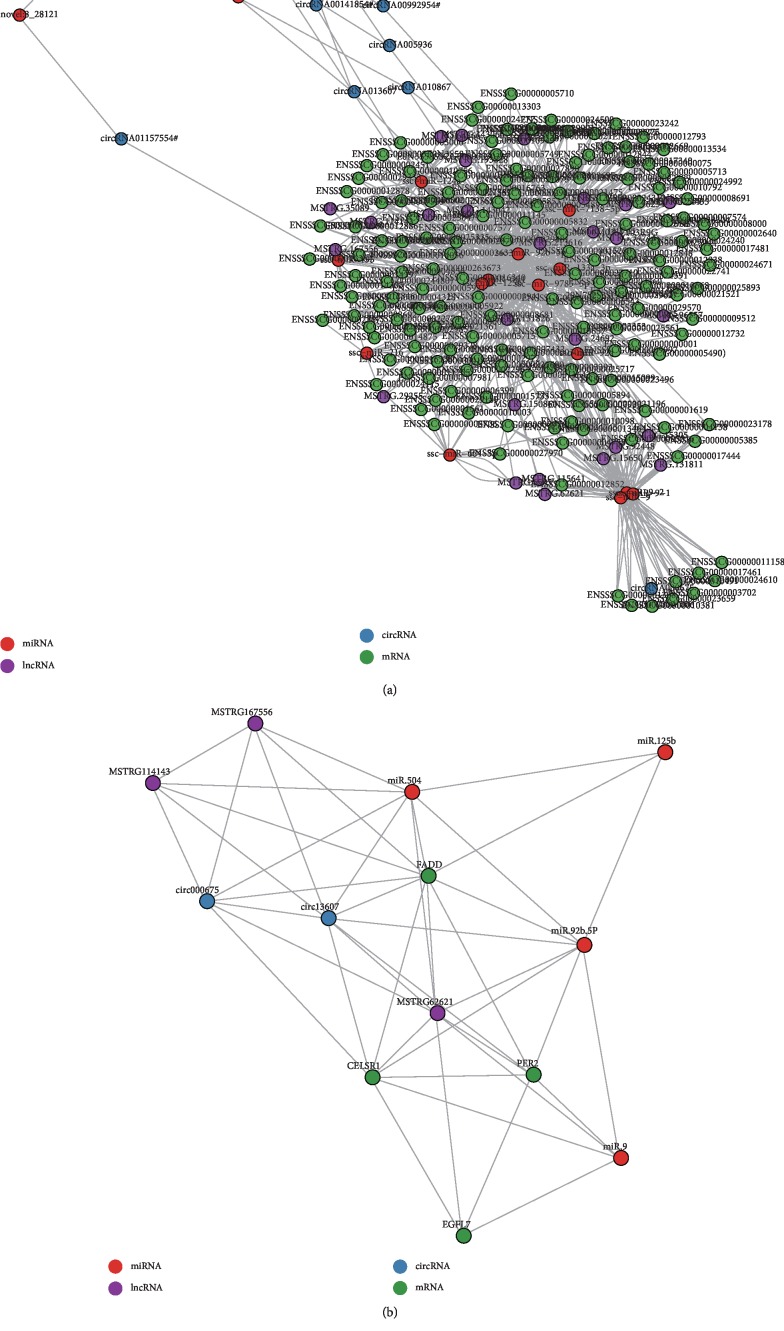
Competing endogenous RNA (ceRNA) network of ovarian aging. (a) The ceRNA network was based on miRNA-mRNA, miRNA-lncRNA, and miRNA-circRNA interactions with microRNA response elements (MREs). (b) Coexpression network between four RNAs classes. Coexpressed RNAs pairs were identified using strict screening criteria (Pearson's correlation coefficients >0.85 or <−0.85, *P* < 0.01).

Based on the competing endogenous RNA (ceRNA) hypothesis [63], ceRNA networks were constructed among these differentially expressed RNAs, which shared a common binding site of the MRE (Figure 5(a) and Supplementary [Supplementary-material supplementary-material-1]). The network consists of a large number of interrelated RNAs that include 20 miRNAs, 170 mRNAs, 27 lncRNAs, and eight circRNAs, and miRNAs played a primary role by targeting other RNA species with MRE in the network. At the upper part of the network, all novel miRNAs were only targeted by circRNAs; however, at the lower part of the network, three miRNAs (miR-9, miR-9-1, and miR-9-2) could simultaneously target nine mRNAs and one circRNA, respectively. In the center of the network, several miRNAs were found including miR-125b, miR-504, and miR-92b-5P, suggesting important roles during the ovarian aging cycle. In fact, previous studies showed that these miRNAs exert important effects on ovarian function. For instance, miR-125b was a negative regulator for p53-induced apoptosis [64], promoted both the proliferation and migration of type II endometrial carcinoma cells [65], and suppressed ovarian cancer cell proliferation [66]. MiR-504 stimulated cancer cell apoptosis and hindered cancer cell proliferation [67, 68]. MiR-92 b promoted the growth of non-small-cell lung cancer cells [69] and controlled the G1/S cell cycle phase in human embryonic stem cells [70]. In addition, miR-9 can act as a candidate tumor suppressor gene in recurrent ovarian cancer [62] and regulate neural development [71]. Due to the important effects of the four miRNAs on ovarian function, their corresponding target genes were further screened and identified. Furthermore, previous studies showed that these target genes of the four miRNAs, which included Fas Associated Via Death Domain (*FADD*), Period Circadian Clock 2 (*PER2*), Cadherin EGF LAG Seven-Pass G-Type Receptor 1 (*CELSR1*), and EGF-Like Domain Multiple 7 (*EGFL7*) were related to reproductive deficits [72], embryogenesis [73], and differentiation of embryonic stem cells [74].

The ceRNA network based on MRE prompted the further analysis of the coexpression relationship among these RNAs. Therefore, found miRNAs (miR-125b, miR-504, miR-92b-5P, and miR-9) were selected that are related to ovarian function. Their target genes (*FADD*, *EGFL7*, *PER2*, and *CELSR1*), target lncRNAs (MSTRG62621, MSTRG114143, and MSTRG167556), and target circRNAs (circ000675 and circ13607) were used to construct a coexpression network using Pearson's correlation coefficient according to the differentially expressed RNA data (Figure 5(b) and Supplementary [Supplementary-material supplementary-material-1]). Compared to the network based on MRE, the coexpression network showed more complex relationships among these RNAs. Firstly, circ13607 was correlated to the miR-504 expression level although they did not share the same MRE. The coexpression relationship could be mediated by *FADD*, MSTRG.114143, and miR-92b-5p because miR-504 and miR-92b-5p can target MSTRG.114143 and *FADD* with MRE. Secondly, miR-92b-5p correlated to *FADD*, *PER2*, and *CELSR1* expression levels; however, miR-92b-5p and *PER2* did not share the same MRE, suggesting that other mediators might mediate the coexpression relationship between both. The coexpression relationship between *PER2* and miR-92b-5p were mediated in two ways: *CELSR1*, *PER2*, and circ13607 competed with miR-125b and *CELSR1* and circ13607 competed with miR-92b-5p.

To verify the reliability of the obtained RNA-sequence data, the expression levels of all RNAs were verified by q-PCR and the results are shown in Figure 5(b). This comparison confirmed that the q-PCR results for these RNAs expression were in accordance with the RNA-sequence data (Supplementary [Supplementary-material supplementary-material-1]B–E).

## 4. Discussion

### 4.1. Dynamic Changes of DNA Epigenetic Marks in Ovarian Aging

This study described a combined DNA methylation and transcriptome analysis of OP ovaries and YP ovaries and found progressive changes of age-differential methylation and gene expression landscapes during ovarian aging. DNA methylation modification is a key epigenetic mechanism involved in the vital processes of mammalian development, such as imprinting, transcriptional silencing, and X-chromosome inactivation [47, 75], as well as in the regulation of ovarian cancer and diseases such as PCOS and POI [10] and oocyte aging [11]. This study first reports the DNA methylome profiles during natural ovarian aging and identified several differentially methylated genes involved in embryonic development, death effector domain binding, and cell apoptotic signaling pathways, which indicated that DNA methylation played a vital role during ovarian aging. Similarly, DNA methylation among other tissues was also found to be responsible for tissue aging. For example, in human aging brain tissue, DMR genes were linked to neurodevelopment-related terms, comprising the regulation of neurogenesis and cell projection organization [76]; however, DNA methylation in porcine aging skeletal muscle was reported to be involved in the protein degradation process and also responsible for muscular atrophy [51]. Several cell death pathway genes such as cysteinyl aspartate proteases 10 (*CASP10*) and *CFLAR*, which played important roles in apoptosis [77], were found in the current DMRs data. This further indicated that oocyte apoptosis led to the depletion of ovarian reserves during ovarian aging [78].

### 4.2. Differentially Expressed Transcriptome Related to the Ovarian Aging Cycle

In other genetic systems, it has been reported that several RNA species play critical roles in the regulation of ovarian physiology, and these RNA species are involved in protein-coding RNAs [79–81] and noncoding RNAs such as miRNAs [12–14] and lncRNAs [15, 16]. The current study presents the global transcriptome expression profile of ovarian aging and identified a large number of differentially expressed RNAs. Genes associated with these differentially expressed RNAs were mainly involved in the ovarian aging cycle such as cell apoptosis, reproduction and fertilization process, embryonic development, ovarian cumulus expansion, and ovulation cycle.

Furthermore, several species of RNA could operate as ceRNA, which communicated with and regulated each other by using MREs as language and competed for binding to common miRNAs, which affected the stability of target genes or the translational activity [63]. In this experiment, ceRNA networks were developed based on MRE and coexpression levels and the ceRNA networks were mainly involved in essential ovary developmental biological processes. For example, as a ceRNA, *FADD* and *PER2* competed for binding to miR-92b-5p, thus affecting the expression level of target genes. MiR-92b-5p might promote lung cancer and non-small-cell lung cancer cell growth [69] and controlled the G1/S cell cycle phase in human ES cells [70]; furthermore, *FADD* and *PER2* genes were reported to be related to reproductive deficits [72] and embryogenesis [73]. Understanding the crosstalk between these novel RNAs will provide insights into the regulatory networks of genes with implications in ovarian aging.

### 4.3. Model Organism for Human Ovarian Aging or Disease Research

These conclusions promote the further research on pigs as model animal for ovarian aging or research of human diseases. Previous research identified genes that were differentially expressed in an age-dependent manner using microarray analysis of mouse oocytes, ovary, and ovarian surface epithelial cells as well as human oocytes [79, 82–86]. Although microarray analysis has been performed in porcine to identify the mechanisms involved in the muscle and brain aging [51, 87], the aging process of ovaries has not been specifically investigated. Here, YP (180 days old) and OP (8 years old) were studied to search the DNA methylation and transcriptional expression changes in ovaries during the aging process. Although this study has its limitations, since whole ovaries were sampled, which contain multiple cell types [86, 88], hundreds of differentially methylated and expressed genes were identified to be related to ovarian aging, and these global DNA methylation and transcriptome expression profiles of pig ovaries of different ages constitute a useful resource.

In future, investigations of age-related differential DNA methylation and gene expression in individual cell types are required. Furthermore, this study only selected two age groups and limited samples. Studying pigs of additional successive ages and incorporating more samples are compulsory to further understand the differences in epigenetic modifications related to age, in addition to complicated mechanisms underlying the aging process. Moreover, in addition to providing novel evidence for biomedical studies, genomic/epigenomic searches on pigs can be helpful to discover the underlying molecular basis of the economic traits of pigs. Such knowledge can be used to progress the efficiency of artificial selection to produce more piglets.

## 5. Conclusions

Microarray analyses were performed to identify the differentially methylated and expressed genes in ovary tissues of OP and YP. Through GO enrichment analyses and the construction of ceRNA networks, the functions of differentially methylated and expressed genes, correlated pathways, and mutual regulatory relationships were analyzed between coding and noncoding genes. The obtained results contribute to the understanding of the aging process in ovaries and provide a basis for the future search for the molecular mechanisms of ovarian aging.

## Data Availability

All data generated as part of this study are included in this published article and its supplementary information files. Requests for the raw data should be sent to the corresponding author.

## Supplementary Materials

Supplementary Materials Table S1: primer sequences for q-PCR. Table S2: summary of sequence data and read-alignment statistics. Table S3: percentage of cytosine methylation after extraction. Table S4: association analysis of regulated genes between both ovarian development stages. Table S5: overlap between different methylation regions and differentially expressed genes. Figure S1: DNA methylation levels of CpG CHG and CHH on each chromosome. Figure S2: Pearson's correlation between DNA methylation level and chromosomal features. (A) Scatter plot and trend line (Pearson's correlation), indicating the correlation between the chromosome length and methylation level. (B) Scatter plot and trend line (Pearson's correlation), indicating the correlation between the chromosome GC content and methylation level. (C) Scatter plot and trend line (Pearson's correlation), indicating the correlation between the gene number in chromosome and methylation. (D) Scatter plot and trend line (Pearson's correlation), indicating the correlation between the chromosome CGI ratio and methylation level. (E) Scatter plot and trend line (Pearson's correlation), indicating the correlation between the chromosome repeat number and methylation level. Figure S3: verification of whole genome bisulfite and RNA sequencing data. (A) DNA methylation was verified via bisulfite sequencing PCR. The expression levels of mRNAs (B), miRNAs (C), lncRNAs (D), and circRNAs (E) were verified via q-PCR and adjusted by three endogenous control genes (porcine GAPDH, ACTB, and U6 snRNA). Figure S4: DNA methylation levels and RNA expression levels on each chromosome. Supplementary Database S1: summary and function enrichment of different methylation regions. Supplementary Database S2: identified mRNAs, miRNAs, lncRNAs, and circRNAs in all samples. Supplementary Database S3: differentially expressed mRNAs, miRNAs, lncRNAs, and circRNAs between both stages. Supplementary Database S4: GO analysis of differentially expressed RNAs and function overlap. Supplementary Database S5: targeted by MRE and coexpression of mRNAs, miRNAs, lncRNAs, and circRNAs.
