# Supplementary material for: Dynamic Changes of DNA Methylation and Transcriptome Expression in Porcine Ovaries during Aging
Source: Biomed Res Int. 2019 Oct 30;2019:8732023. doi: 10.1155/2019/8732023 (PMC6874880; doi:10.1155/2019/8732023)
Supplement: Supplementary Materials — Table S1: primer sequences for q-PCR. Table S2: summary of sequence data and read-alignment statistics. Table S3: percentage of cytosine methylation after extraction. Table S4: association analysis of regulated genes between both ovarian development stages. Table S5: overlap between different methylation regions and differentially expressed genes. Figure S1: DNA methylation levels of CpG CHG and CHH on each chromosome. Figure S2: Pearson's correlation between DNA methylation level and chromosomal features. (A) Scatter plot and trend line (Pearson's correlation), indicating the correlation between the chromosome length and methylation level. (B) Scatter plot and trend line (Pearson's correlation), indicating the correlation between the chromosome GC content and methylation level. (C) Scatter plot and trend line (Pearson's correlation), indicating the correlation between the gene number in chromosome and methylation. (D) Scatter plot and trend line (Pearson's correlation), indicating the correlation between the chromosome CGI ratio and methylation level. (E) Scatter plot and trend line (Pearson's correlation), indicating the correlation between the chromosome repeat number and methylation level. Figure S3: verification of whole genome bisulfite and RNA sequencing data. (A) DNA methylation was verified via bisulfite sequencing PCR. The expression levels of mRNAs (B), miRNAs (C), lncRNAs (D), and circRNAs (E) were verified via q-PCR and adjusted by three endogenous control genes (porcine GAPDH, ACTB, and U6 snRNA). Figure S4: DNA methylation levels and RNA expression levels on each chromosome. Supplementary Database S1: summary and function enrichment of different methylation regions. Supplementary Database S2: identified mRNAs, miRNAs, lncRNAs, and circRNAs in all samples. Supplementary Database S3: differentially expressed mRNAs, miRNAs, lncRNAs, and circRNAs between both stages. Supplementary Database S4: GO analysis of differentially expressed RNAs and function over [file 8732023.f1.zip › 8732023.f1/Supplementary Figures.docx]

| **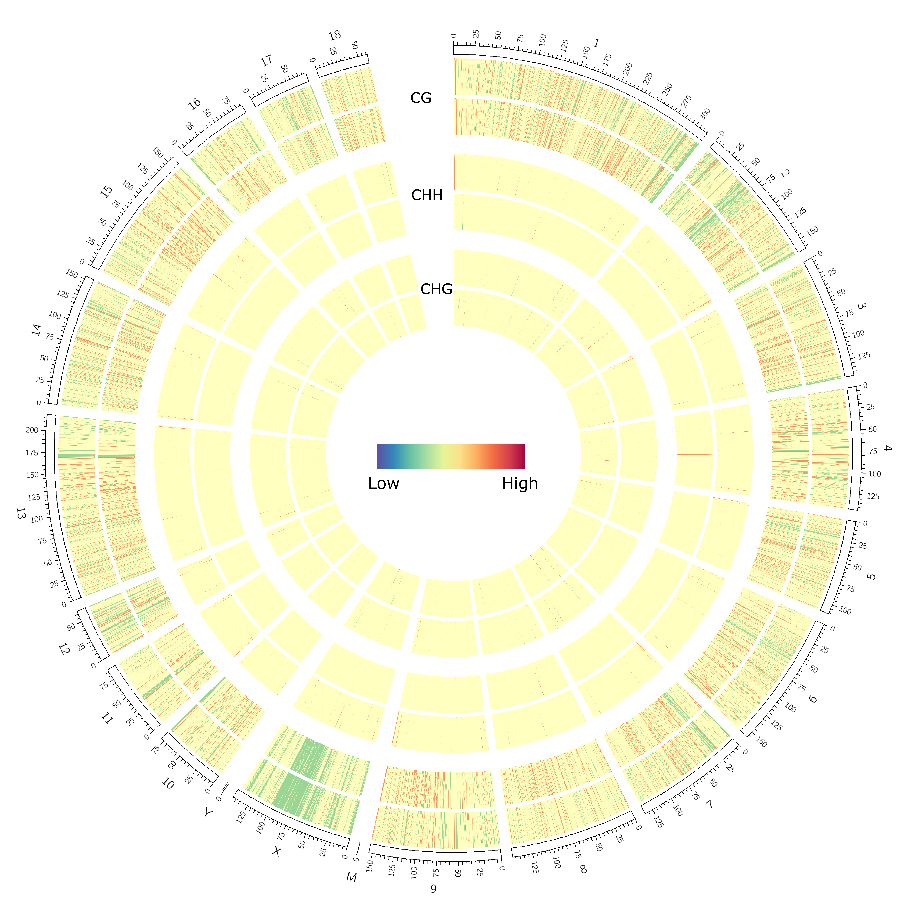**  **Supplementary Figure. S1.** DNA methylation levels of CpG CHG and CHH on each chromosome. In each of the three circles, the outer layer indicates the methylation level of young pigs and the inner layer indicates the methylation level of old pigs, respectively. |
| --- |
|  |

**Supplementary Figures**

| **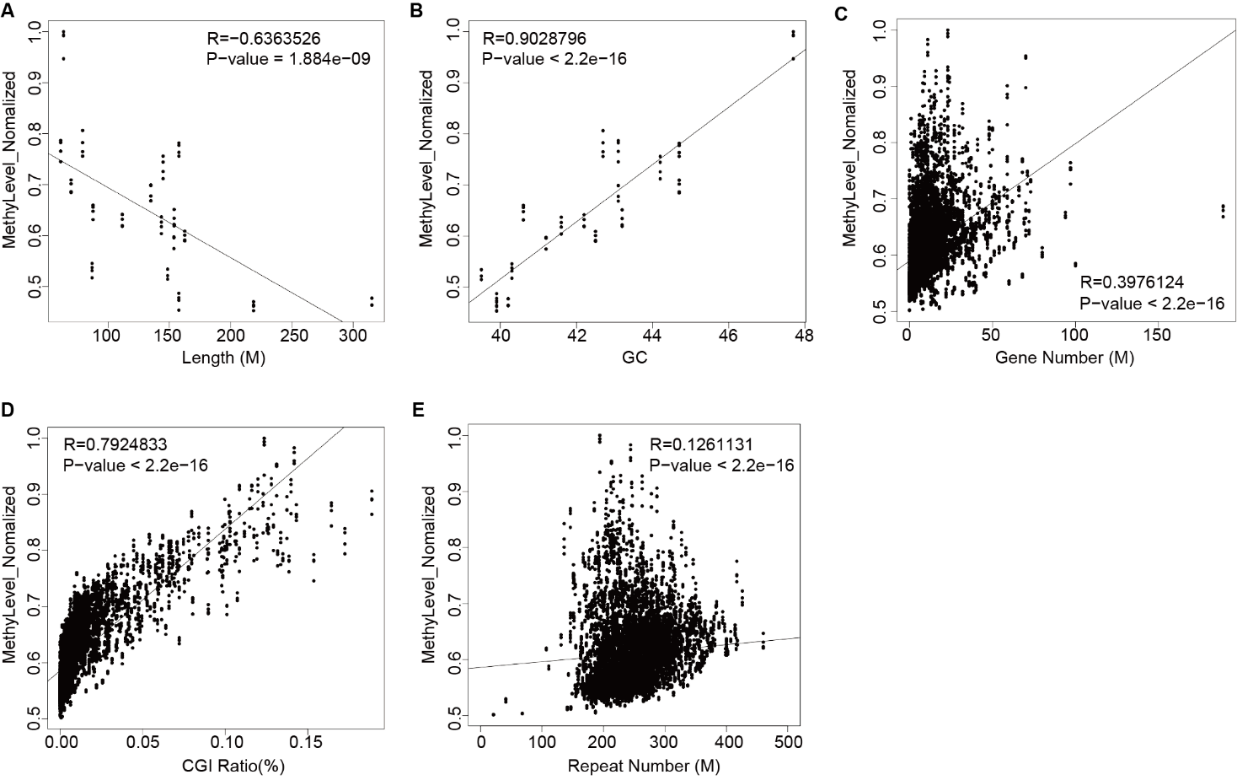**  **Supplementary Figure. S2.** Pearson’s correlation between DNA methylation level and chromosomal features. **(A)** Scatter plot and trend line (Pearson’s correlation) showing the correlation between the chromosome length and methylation level. The line represents a linear regression. **(B)** Scatter plot and trend line (Pearson’s correlation) showing the correlation between the chromosome GC content and methylation level. The line represents a linear fortify. **(C)** Scatter plot and trend line (Pearson’s correlation) showing the correlation between the gene number in chromosome and methylation. The line represents a linear fortify. **(D)** Scatter plot and trend line (Pearson’s correlation) showing the correlation between the chromosome CpG island (CGI) ratio and methylation level. The line represents a linear fortify. **(E)** Scatter plot and trend line (Pearson’s correlation) showing the correlation between the chromosome repeat number and methylation level. The line represents a linear fortify. |
| --- |

| **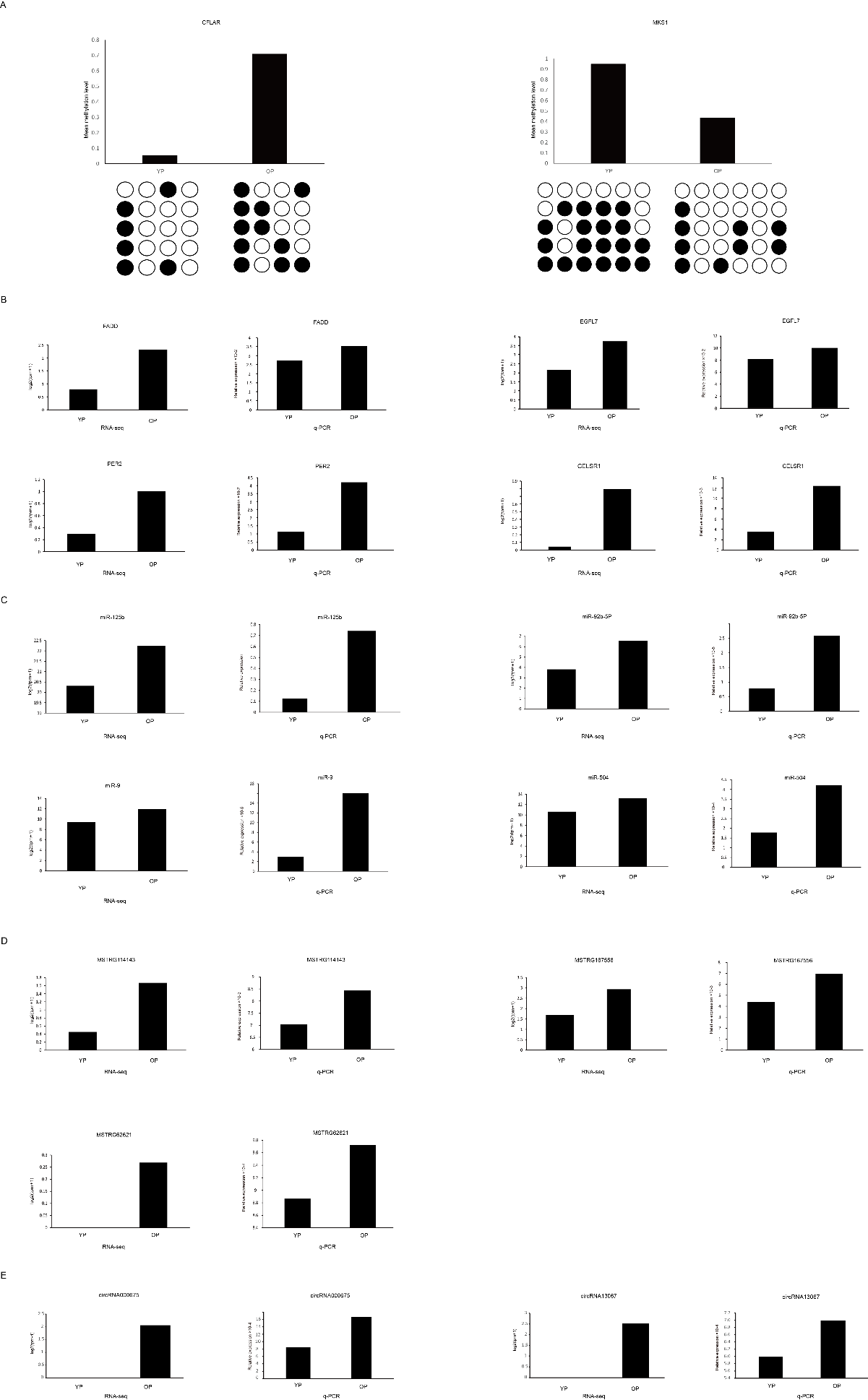**  **Supplementary Figure. S3.** The verification of whole genome bisulfite and RNA sequencing data. **(A)** DNA methylation was verified by bisulfite sequencing PCR (BSP). The expression levels of mRNA (**B**), miRNA (**C**), lncRNA (**D**) and circRNA (E) were verified by qPCR and adjusted by three endogenous control genes (porcine GAPDH, ACTB and U6 snRNA). The 2^-ΔΔCt^ method was used to determine the expression level of objective mRNAs, miRNAs, lncRNAs and circRNA. |
| --- |
| **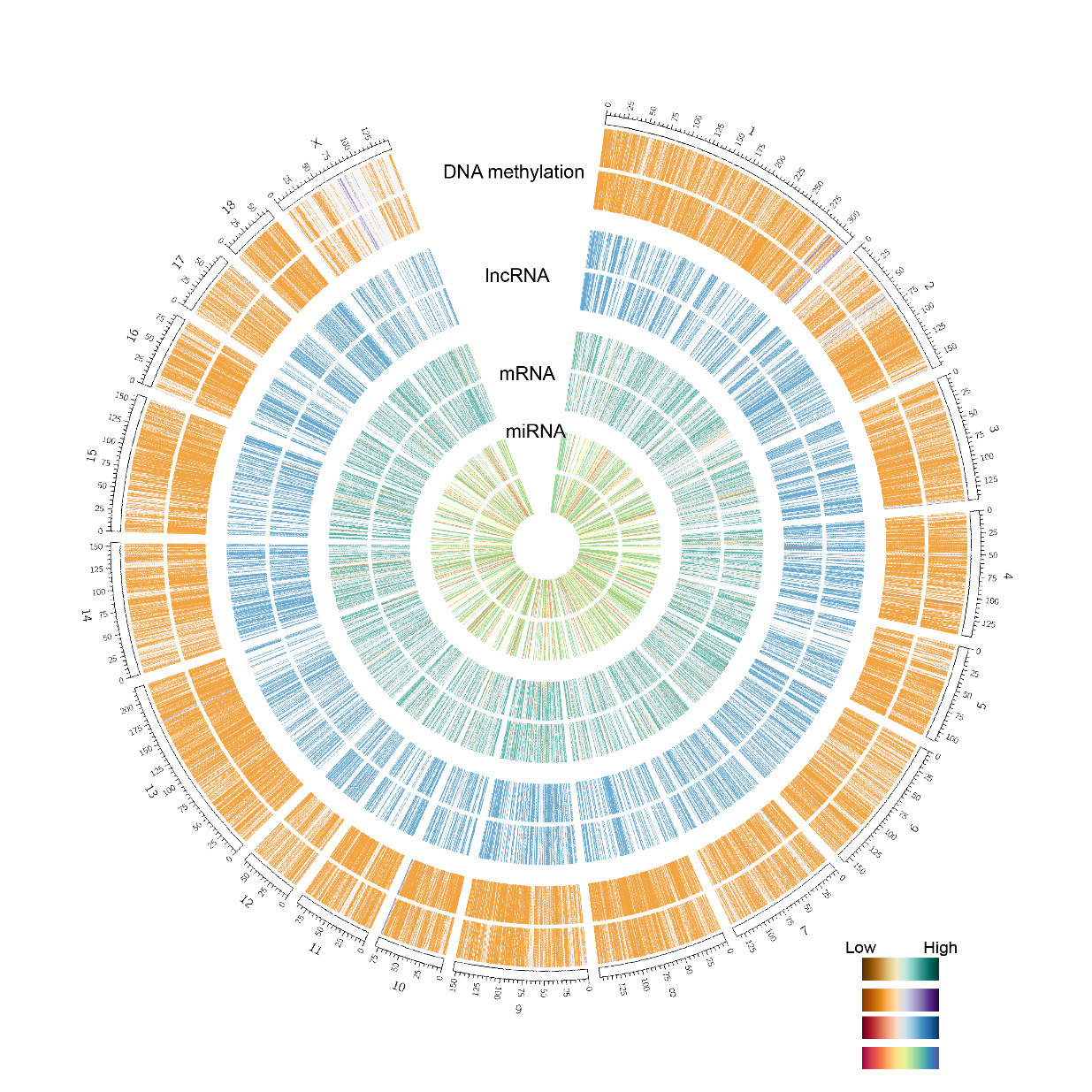**  **Supplementary Figure. S4.** The DNA methylation levels and RNAs expression levels on each chromosome. In each of the three circles, the outer layer indicates the methylation level of young pigs and the inner layer indicates the methylation level of old pigs, respectively. |
