# Supplementary material for: Dynamic Changes of DNA Methylation and Transcriptome Expression in Porcine Ovaries during Aging
Source: Biomed Res Int. 2019 Oct 30;2019:8732023. doi: 10.1155/2019/8732023 (PMC6874880; doi:10.1155/2019/8732023)
Supplement: Supplementary Materials — Table S1: primer sequences for q-PCR. Table S2: summary of sequence data and read-alignment statistics. Table S3: percentage of cytosine methylation after extraction. Table S4: association analysis of regulated genes between both ovarian development stages. Table S5: overlap between different methylation regions and differentially expressed genes. Figure S1: DNA methylation levels of CpG CHG and CHH on each chromosome. Figure S2: Pearson's correlation between DNA methylation level and chromosomal features. (A) Scatter plot and trend line (Pearson's correlation), indicating the correlation between the chromosome length and methylation level. (B) Scatter plot and trend line (Pearson's correlation), indicating the correlation between the chromosome GC content and methylation level. (C) Scatter plot and trend line (Pearson's correlation), indicating the correlation between the gene number in chromosome and methylation. (D) Scatter plot and trend line (Pearson's correlation), indicating the correlation between the chromosome CGI ratio and methylation level. (E) Scatter plot and trend line (Pearson's correlation), indicating the correlation between the chromosome repeat number and methylation level. Figure S3: verification of whole genome bisulfite and RNA sequencing data. (A) DNA methylation was verified via bisulfite sequencing PCR. The expression levels of mRNAs (B), miRNAs (C), lncRNAs (D), and circRNAs (E) were verified via q-PCR and adjusted by three endogenous control genes (porcine GAPDH, ACTB, and U6 snRNA). Figure S4: DNA methylation levels and RNA expression levels on each chromosome. Supplementary Database S1: summary and function enrichment of different methylation regions. Supplementary Database S2: identified mRNAs, miRNAs, lncRNAs, and circRNAs in all samples. Supplementary Database S3: differentially expressed mRNAs, miRNAs, lncRNAs, and circRNAs between both stages. Supplementary Database S4: GO analysis of differentially expressed RNAs and function over [file 8732023.f1.zip › 8732023.f1/Supplementary Tables.docx]

**Supplementary Tables**

| **Supplementary Table S1. Primer sequences for q-PCR** | | |  |
| --- | --- | --- | --- |
| Genes | Forward | Reverse | Tm (°C) |
| CELSR1 | 5'-CACCCTCATCTGGAGCTTTGC-3' | 5'- ATAATGGCGCTTCCTTTGGC-3' | 63 |
| EGFL7 | 5'-TGCCAGACAGATGTGGATGAAT-3' | 5'-CGCTCCTGTTGTGGGGTTT-3' | 63 |
| FADD | 5'-AGTGTCTGACGCCAAGATCGA-3' | 5'-TCCCTCCTGCTGTTCTTCCA-3' | 63 |
| PER2 | 5'-CCAGTCTCCCTGCCACCATTA-3' | 5'-AGGCTTGACCCGTTTGGACTT-3' | 63 |
| GAPDH | 5'- TTCCAGTATGATTCCACCCACG-3' | 5'- CCATTTGATGTTGGCGGGAT-3' | 63 |
| ACTB | 5'-CTGCGGCATCCACGAAACT-3' | 5'- GTGATCTCCTTCTGCATCCTGTC-3' | 63 |
| MSTRG.62621 | 5'-CACGCTGGGTGACCAAATG-3' | 5'-TTGAAGCATAAACAGGGAAGGAG-3' | 63 |
| MSTRG.114143 | 5'-AGAAACCAGGTCACCCAGAGC-3' | 5'-GTAATAGCGTGTAGGAGAAGCAGC-3' | 63 |
| MSTRG.167556 | 5'-TGTGGGTCTCGTGTTCAGCC-3' | 5'-TACCGTGACATAAACAGTGAGCAGT-3' | 63 |
| miR-9 | 5'-GCGTCTTTGGTTATCTAGCTGTATG-3' | Universal primer supplied by kit | 63 |
| miR-92b-5p | 5'-GGGACGCGGTGCAGTGTTA-3' | Universal primer supplied by kit ' | 63 |
| miR-125b | 5'-GCTCCCTGAGACCCTAACTTGTGA-3' | Universal primer supplied by kit ' | 63 |
| miR-504 | 5'-AGACCCTGGTCTGCACTCTATCT-3' | Universal primer supplied by kit ' | 63 |
| U6 | 5'-GGAACGATACAGAGAAGATTAGC-3' | 5'-TGGAACGCTTCACGAATTTGCG-3' | 63 |
| circ000675 | 5'-CCATCTTTTCCACAAGCACG-3' | 5'-GTATACCAAATCAAAAGGACCAGA-3' | 60 |
| circ013607 | 5'-CCAGAATATGGGGTAACCAGGT-3’ | 5'-CAGAACCTGGACGGGACTAC-3' | 60 |
| Note: U6 snRNA, ACTB, GAPDH were used for endogenous control genes to adjust the result of qPCR. | | | |

| **Supplementary Table S2. Summary of sequence data and read-alignment statistics** | | | | |
| --- | --- | --- | --- | --- |
|  | Samples | Clean Reads | Mapped Reads | Mapping Rate |
| DNA methylation | YP-1 | 262,637,380 | 160,363,675 | 61.06% |
|  | YP-2 | 288,708,667 | 191,645,239 | 66.38% |
|  | OP-1 | 180,384,057 | 122,058,091 | 67.67% |
|  | OP-2 | 219,710,200 | 139,838,322 | 63.65% |
| miRNAs libraries | YP-1 | 12,979,459 | 9,844,992 | 75.85% |
|  | YP-2 | 10,894,120 | 8,875,896 | 81.47% |
|  | OP-1 | 11,219,267 | 9,061,296 | 80.77% |
|  | OP-2 | 13,517,349 | 12,232,599 | 90.50% |
| RNA libraries | YP-1 | 68,448,044 | 47,776,816 | 69.80% |
|  | YP-2 | 62,084,328 | 42,902,544 | 69.10% |
|  | OP-1 | 66,623,964 | 49,771,144 | 74.70% |
|  | OP-2 | 102,679,586 | 70,861,325 | 69.01% |
| Note: YP-1and YP-2 mean the sample 1 and sample from young sows in puberty stage, respectively; OP-1 and OP-2 mean the sample 1 and sample 2 from old sows in reproductive exhaustion stage, respectively. | | | | |

| **Supplementary Table S3. Percentage of cytosine methylation after extraction** | | | | |
| --- | --- | --- | --- | --- |
|  | YP-1 | YP-2 | OP-1 | OP-2 |
| Percentage of CpG context | 66.50% | 68.30% | 70.70% | 69.50% |
| Percentage of CHG context | 2.00% | 1.50% | 1.30% | 1.70% |
| Percentage of CHH context | 1.80% | 1.30% | 1.10% | 1.50% |
| Note: YP-1and YP-2 mean the sample 1 and sample from young sows in puberty stage, respectively; OP-1 and OP-2 mean the sample 1 and sample 2 from old sows in reproductive exhaustion stage, respectively. | | | | |

| **Supplementary Table S4. Association analysis of expressed genes between both ovarian development stages** | | | | | | | | | |
| --- | --- | --- | --- | --- | --- | --- | --- | --- | --- |
| YP | Total | 8845 | 1190 | 3430 | 8662 | 939 | 289 | 1331 | 636 |
|  | Rate | 0.349 | 0.047 | 0.135 | 0.342 | 0.037 | 0.011 | 0.053 | 0.025 |
|  | Methylation | + | + | + | + | - | - | - | - |
|  | mRNA | + | + | - | - | + | + | - | - |
|  | miRNA | + | - | - | + | + | - | + | - |
| OP | Total | 9663 | 1285 | 3335 | 7855 | 1016 | 309 | 1243 | 616 |
|  | Rate | 0.382 | 0.0508 | 0.132 | 0.31 | 0.04 | 0.012 | 0.049 | 0.024 |
|  | Methylation | + | + | + | + | - | - | - | - |
|  | mRNA | + | + | - | - | + | + | - | - |
|  | miRNA | + | - | - | + | + | - | + | - |
| YP | Total | 1354 | 1008 | 1278 | 3453 | 147 | 312 | 323 | 232 |
|  | Rate | 0.167 | 0.1243 | 0.158 | 0.426 | 0.018 | 0.038 | 0.04 | 0.029 |
|  | Methylation | + | + | + | + | - | - | - | - |
|  | lncRNA | + | + | - | - | + | + | - | - |
|  | miRNA | + | - | - | + | + | - | + | - |
| OP | Total | 1465 | 1173 | 1115 | 3347 | 185 | 367 | 280 | 175 |
|  | Rate | 0.181 | 0.1447 | 0.138 | 0.413 | 0.023 | 0.045 | 0.035 | 0.022 |
|  | Methylation | + | + | + | + | - | - | - | - |
|  | lncRNA | + | + | - | - | + | + | - | - |
|  | miRNA | + | - | - | + | + | - | + | - |
| Note: YP means young sows in puberty stage and OP means the old sows in reproductive exhaustion stage. The numbers and percentages of genes in each possible combination of expression are calculated. “+”represents methylated/expressed while “-” represents not methylated/not expressed. | | | | | | | | | |

**Supplementary Table S5. Overlap between different methylation regions and differentially expressed genes**

| mRNA ID | Chromosome | Start | End | Location | Hypermethylated  Hypomethylated | Up or down  expressed |
| --- | --- | --- | --- | --- | --- | --- |
| ENSSSCG00000026570 | chr4 | 86682776 | 86682849 | Promoter | ↓ | up |
| ENSSSCG00000009000 | chr8 | 79314978 | 79315196 | Genebody | ↑ | up |
| ENSSSCG00000015402 | chr9 | 108351134 | 108351432 | Genebody | ↑ | down |
| ENSSSCG00000000082 | chr5 | 5948245 | 5948301 | Genebody | ↑ | up |
| ENSSSCG00000016848 | chr16 | 24705654 | 24705786 | Genebody | ↑ | down |
| ENSSSCG00000028471 | chr15 | 50821506 | 50821760 | Genebody | ↑ | up |
| ENSSSCG00000004101 | chr1 | 18488960 | 18489504 | Genebody | ↓ | down |
| ENSSSCG00000029688 | chr15 | 50736155 | 50736331 | Genebody | ↓ | up |
|  |  | 50821506 | 50821760 | Genebody | ↑ |  |

Note: ↑is represented hypermethylated; ↓is represented hypomethylated.
